# Commentary: Emotional freedom techniques for treating post traumatic stress disorder: an updated systematic review and meta-analysis

**DOI:** 10.3389/fpsyg.2024.1308687

**Published:** 2024-02-22

**Authors:** Rory A. Pfund, Cassandra L. Boness, David F. Tolin

**Affiliations:** ^1^Department of Psychology, Tennessee Institute for Gambling Education & Research, Memphis, TN, United States; ^2^Department of Psychology, The University of Memphis, Memphis, TN, United States; ^3^Center on Alcohol, Substance Use, and Addictions, University of New Mexico, Albuquerque, NM, United States; ^4^Anxiety Disorders Center, Institute of Living, Hartford, CT, United States; ^5^Department of Psychiatry, Yale University School of Medicine, New Haven, CT, United States

**Keywords:** pseudoscience, ethics, emotional freedom techniques, thought field therapy, acupressure, meta-analysis

## Introduction

Ioannidis (2016) has illustrated that the number of meta-analyses has proliferated over the last several decades. Indeed, the number of meta-analyses published annually has increased exponentially in this time (Papatheodorou, 2019), and the number of meta-analyses now likely exceeds the number of randomized trials published annually (Ioannidis, 2016). This mass production of meta-analyses is potentially harmful if a given meta-analysis's contributions and methods are not clearly differentiated from existing meta-analyses, because publishing redundant results can give a false impression that the literature is more robust than it actually is. This problem is especially concerning given that meta-analyses sit atop the “hierarchy of evidence” and carry substantial weight in decision making about evidence-based medicine and psychology (American Psychological Association, 2006; Murad et al., 2016).

Stapleton et al. (2023) meta-analysis entitled “Emotional freedom techniques for treating post traumatic stress disorder: an updated systematic review and meta-analysis,” used meta-analytic methods that notably overlap with a prior meta-analysis on emotional freedom techniques (EFT) for posttraumatic stress disorder (PTSD) by Sebastian and Nelms (2017). Although Stapleton et al. (2023) wrote that the prior meta-analysis warranted an update due to the “increase in research and time elapsed since the initial meta-analysis on EFT for PTSD” (p. 4), Table 1 shows that five of the six articles (83.3%) in the Stapleton meta-analysis were included in the Sebastian and Nelms meta-analysis, and the sixth article was published before the Sebastian and Nelms meta-analysis. Surprisingly absent is a reason why two primary studies (Church et al., 2012, 2018) included in the Sebastian and Nelms meta-analysis were not included in the Stapleton meta-analysis, as both primary studies appear to meet the only stated inclusion criteria of “an RCT investigating the use of EFT for treating the symptoms of trauma or PTSD” (Stapleton et al., 2023; p. 4). Thus, Stapleton et al. did not sufficiently support their claim that the elapsed time corresponded to an increase in research on EFT for PTSD.

**Table 1:** Overlap in studies between Stapleton and Sebastian and Nelms.

	**Meta-analysis**	
**Study**	**Stapleton et al. (** **2023** **)**	**Sebastian and Nelms (** **2017** **)**
Al-Hadethe et al. (2015)	Included	Not included
Church et al. (2012)	Not included	Included
Church et al. (2013)	Included	Included
Church et al. (2016)	Included	Included
Church et al. (2018)	Not included	Included
Geronilla et al. (2016)	Included	Included
Karatzias et al. (2011)	Included	Included
Nemiro and Papworth (2015)	Included	Included

Stapleton et al.'s meta-analytic methods were also virtually identical to those used in the Sebastian and Nelms meta-analysis. Both meta-analyses used standard methods to calculate a Cohen's *d* effect size from pre-treatment and post-treatment data from the EFT study conditions. A major difference in these two meta-analyses was that Stapleton et al. (2023) additionally calculated a Cohen's *d* effect size representing post-treatment data between EFT and waitlist conditions, but this was never acknowledged as a methodological strength or difference from the Sebastian and Nelms meta-analysis.

In addition to these methodological overlaps, Stapleton et al. stated that the studies included in their meta-analyses were randomized controlled trials “investigating the use of EFT for treating the symptoms of trauma or PTSD” (p. 4). However, they acknowledged in their limitations section that the “trials included in this meta-analysis all employed self-report measures to evaluate the presence of PTSD and reduction of symptoms” (p. 8). Such an approach to evaluate the presence of PTSD and its symptoms violates foundational practice in clinical psychology that requires an integration of multiple datapoints to warrant diagnosis, including a diagnostic clinical interview. The reliance on self-reported diagnoses and symptoms without the support of a clinical interview and other assessments prevents the authors from claiming that EFT affected PTSD or its symptoms.

Stapleton et al. (2023) made multiple inaccurate statements about EFT and its evidence base. In the introduction, the authors stated that EFT “utilizes techniques from both Cognitive Behavioral Therapy (CBT) and Prolonged Exposure therapy (PE)” (p. 2). This statement is inaccurate, considering that EFT has been repeatedly identified as a pseudoscientific and discredited therapy, and the proposed techniques in EFT do not actually resemble elements from CBT or PE (Norcross and Koocher, 2006; Boness et al., 2023). In the discussion, Stapleton et al. (2023) stated that the results of their meta-analysis indicated that EFT met the American Psychological Association's standards for empirically supported treatments, and that the current meta-analysis “demonstrated Clinical EFT to be an effective evidence-based treatment for PTSD” (p. 9). Both statements are misleading: EFT is not listed on the APA's website as an empirically supported treatment for treating PTSD or any other psychological disorder (www.psychologicaltreatments.org), and multiple independent research teams have discredited the methodological quality and conclusions of randomized controlled trials and meta-analyses on EFT (McCaslin, 2009; Bakker, 2013; Spielmans et al., 2020; Spielmans and Rosen, 2022; Boness et al., 2023). Thus, EFT does not actually meet APA Division 12 (Society of Clinical Psychology)'s standards for empirically supported treatments.

Stapleton et al. (2023) made at least one other inaccurate statement in their declaration of conflicts of interest. The first author declared that “she may lead clinical research trials in the topic.” Without context, it is not entirely clear what is the “topic.” We assume that the first author intends the topic to be EFT, as they write that the first author “does not conduct trials in the area of PTSD” on page 9. Furthermore, this conflict is understated as the first author writes that her “most significant contribution in her research life has been to lead world-first randomized clinical trials investigating Emotional Freedom Techniques” and described the results of that trial as “outstanding” (https://www.petastapleton.com/about). On that same webpage, the first author offers a paid EFT program titled “Emotional Freedom Techniques for Weight Management.” We are concerned that the first author did not disclose that they lead paid EFT trainings because the *Frontiers in Psychology* conflict of interest policy asks authors to declare “other relationships or activities that readers could perceive to have influenced, or that give the appearance of potentially influencing… submitted work.”

Finally, we wonder why a paper on EFT warrants publication in a psychology journal. EFT is predicated on mechanisms that are derived not from psychological science but rather from traditional Chinese medicine and theories of acupuncture (Ma et al., 2016). On page 3, Stapleton et al. themselves describe that “the stimulation of acupuncture points is the primary somatic ingredient of EFT,” and “a core concept of acupuncture is that stimulating electrically sensitive points on the skin sends impulses to related organs along ‘energy pathways' known as meridians.” To our knowledge, EFT is not compatible with any branch of science (Bakker, 2013).

For all the above reasons, we question the contribution of Stapleton et al. (2023) meta-analysis. We also believe it is harmful to individuals seeking treatment for PTSD, detrimental to the scientific understanding of PTSD and its treatment, and inconsistent with ethical practice in professional psychology. Individuals seeking treatment should consult the APA Division 12 website that currently supports CBT, PE, and cognitive processing therapy for PTSD (https://div12.org/treatments/?_sfm_related_diagnosis=8142). Principle C of the APA Ethics Code, Integrity, states that “Psychologists seek to promote accuracy, honesty, and truthfulness in the science, teaching, and practice of psychology” (p. 3–4). Stapleton et al.'s meta-analysis represents an inaccurate representation of PTSD treatments.

## Author contributions

RP: Conceptualization, Writing – original draft, Writing – review & editing. CB: Conceptualization, Writing – original draft, Writing – review & editing. DT: Conceptualization, Writing – original draft, Writing – review & editing.
